# Genetic analysis of Thailand hantavirus in *Bandicota indica *trapped in Thailand

**DOI:** 10.1186/1743-422X-3-72

**Published:** 2006-09-05

**Authors:** Jean-Pierre Hugot, Angelina Plyusnina, Vincent Herbreteau, Kirill Nemirov, Juha Laakkonen, Åke Lundkvist, Yupin Supputamongkol, Heikki Henttonen, Alexander Plyusnin

**Affiliations:** 1 OSEB, UMR 5202 du CNRS, Muséum National d'Histoire naturelle, Paris, France; 2 Institut de Recherche pour le Développement, Paris, France; 3 Department of Virology, Haartman Institute, University of Helsinki, Finland; 4 Swedish Institute for Infectious Disease Control, Stockholm, Sweden; 5 Finnish Forest Research Institute, Vantaa, Finland; 6 Siriraj Hospital, Bangkok, Thailand

## Abstract

Sixty one tissue samples from several rodent species trapped in five provinces of Thailand were examined for the presence of hantaviral markers by enzyme-immunoassay and immunoblotting. Four samples, all from the great bandicoot rat *Bandicota indica*, were confirmed positive for the hantaviral N-antigen. Two of them were trapped in Nakhon Pathom province, the other two in Nakhon Ratchasima province, approximately 250 km from the other trapping site. When analysed by RT-nested PCR, all four rodents were found positive for the hantaviral S- and M-segment nucleotide sequences. Genetic analysis revealed that the four newly described wild-type strains belong to Thailand hantavirus. On the phylogenetic trees they formed a well-supported cluster within the group of Murinae-associated hantaviruses and shared a recent common ancestor with Seoul virus.

## Background

Hantaviruses (genus *Hantavirus*, family *Bunyaviridae*) are robo (from **ro**dent-**bo**rne) viruses that cause hemorrhagic fever with renal syndrome (HFRS) in Eurasia and hantavirus (cardio)pulmonary syndrome (HPS) in the Americas [1-3]. In nature, hantaviruses are carried by rodents of family *Muridae*, and each hantavirus species is predominantly associated with a unique rodent host species. Transmission of the virus to humans occurs by inhalation of virus-infected aerosols from excreta of persistently infected animals. Currently three groups of hantavirus species are recognized [3-5]. The first group is associated with Murinae rodents (mice and rats of the Old World). The hantaviruses that belonged to the second group are carried by Sigmodontinae rodents (mice and rats of the New World). The third group is associated with Arvicolinae rodents (voles and lemmings of the north hemisphere) and includes viruses from Europe, Asia and North America. In addition to these three groups, the list of hantaviral species includes Thottapalayam, so far the only hantavirus found in association with a shrew, *Suncus murinus *[6].

Since hantaviruses have been isolated from Murinae rodents in North Asia and Europe, the association with this particular group of hosts questions the presence of hantaviruses in other parts of the World, and particularly in South East Asia from where murine rodents are considered to originate and where more than 35 species of Murinae rodents are living [7]. Several hantaviruses have been recorded from South-East Asia, particularly: THAIV discovered in 1994 [8] in Thailand from a great bandicoot rat, *Bandicota indica*; and several hantavirus like isolated in Cambodia from *Rattus rattus *and *R. norvegicus *[9]. Also, serological surveys carried out to detect evidence of hantavirus in human populations or in wild rodents, revealed positive samples in Thailand and Cambodia [9-12]. From these preliminary results and after confirmation of a first human case in Thailand [13] several questions arise: What is the genetic diversity of the hantaviruses in South-East Asia? What are the relationships of the South Asian hantaviruses with the others? What is the real importance of hantaviruses for human health in this part of the World? The answers to these questions clearly deal with the hantavirus biodiversity and phylogeny [4,5,14]. They also suppose that coordinated investigations might relate the distribution of the hantaviruses in human populations and in different rodent species.

The first aim of this study was to examine a set of tissue samples from several rodent species trapped in Thailand, for the presence of hantaviral markers. Since the hantaviral N-protein antigen was detected in samples from *B. indica*, it was decided to attempt a recovery of viral genome sequences (S and M segments) from the antigen-positive tissue samples and to perform a (phylo)genetic analysis using these new data. So far, no complete THAIV S-sequence has been described in the literature [1] but while this work was in progress a complete THAIV S-sequence was deposited to Genbank. This sequence belongs to a cell culture isolate 741, originating from Thailand. Thus, our data presented an opportunity to compare the newly recovered sequences of the wild-type THAIV strains with that of a regular THAI isolate.

## Materials and methods

### Trapping/collection

Rodents were collected since 2004 during several field studies in the following provinces of Thailand: Nakhon Ratchasima, Sakhon Nakhon, Phrae, Nakhon Pathom and Loei. Trapping was focused on species living in proximity to humans: domestic and peridomestic species, *Rattus exulans*, *R. rattus*, *R. norvegicus*, and the main wild species occurring in agricultural areas, *Bandicota indica *and *B. savilei*. The study was conducted in agricultural areas including rice-growing rural villages either in seasonally flooded or non-flooded lands. Trapping and processing were performed according to established safety recommendations [15]. Animals were collected early in the morning and transferred to a field laboratory. Geographical coordinates of the trapping places were systematically recorded. Species identification was done using a regional taxonomic identification key [7]. Animals were measured, weighted and pictured. Serum samples and organs were stored in cryovials at -70°C.

### Screening of rodent samples

Rodent lung tissue samples were screened by immunoblotting, for the presence of hantaviral N-antigen as described earlier [16]. In brief, small chips of tissue (approximately 100 mg) were placed into 500 mkl of Laemmli sample buffer and homogenized by sonication. Aliquots of 10 mkl were separated by electrophoresis in 10% sodium dodecyl sulphate-polyacrylamide gels and blotted with rabbit polyclonal antibody raised against Dobrava virus. Goat anti-rabbit antibodies conjugated with the horse radish peroxidase (Dako, Glostrup, Denmark) were used as secondary antibodies. A confirmatory immunoblotting was performed with the rat anti-SEOV antiserum [17]; in this case, rabbit anti-rat antibodies conjugated with the horseraddish peroxidase (Dako, Glostrup, Denmark) were used as secondary antibodies.

### RNA isolation, reverse transcription (RT)-polymerase chain reaction (PCR) and sequencing

RNA was purified from N antigen- positive samples with the TriPure reagent (Behringer Maannheim) following the manufacturer's instructions. Approximately 100 mg- piece of each lung tissue sample was ground in 1 ml of the TriPure reagent and subjected to RNA extraction. RT-PCR of the entire hantaviral S segment was performed essentially as described previously [18,19]. Partial sequences of the S segment (nt 389–946) and the M segment (nt 2021–2303) from wild-type THAIV strains were obtained by RT-nested PCRs (sequences of primers are available upon request). PCR-amplicons were gel-purified using QIAquick Gel Extraction -kit (QIAGEN). PCR-amplicon containing the entire S-sequences was cloned using the pGEM-T cloning kit (Promega) and the plasmids were purified with the QIAprep kit (QIAgen). PCR-amplicons containing the partial S- and M-sequences were gel-purified using QIAquick Gel Extraction -kit (QIAGEN). The plasmids and PCR-amplicons were sequenced automatically using either ABI PRISM™ Dye Terminator or ABI PRISM™ M13F and M13R Dye Primer sequencing kits (Perkin Elmer/ABI, NJ). Multiple nucleotide and amino sequence alignments were prepared manually using SeqApp 1.9a169 sequence editing program. Hantavirus sequences used for comparison were recovered from the Gene Bank.

### Phylogenetic analysis

To infer phylogenies, the PHYLIP program package [20] was used first. 500 bootstrap replicates generated for complete coding sequences of the S segment, as well as partial sequences of the S segment and the M segments (Seqboot program) were fed to the distance matrice algorithm (Dnadist program, with the F84-model for nucleotide substitution). Distance matrices were analysed with the Fitch-Margoliash tree-fitting algorithm (Fitch program); the bootstrap support values were calculated with the Consense program. The nucleotide sequence data were also analysed with the Tree-Puzzle program [21]. The program implements a fast tree-searching algorithm (quartet puzzling) that allows reconstruction of phylogenetic trees by maximum likelihood. All trees were calculated with 10000 puzzling steps using Hasegawa-Kishino-Yano model of nucleotide substitutions. The transition/transversion ratio and the nucleotide frequencies were estimated from the data set. Uniformal model of rate heterogeneity across sites was applied.

## Results

### Screening of rodents for the presence of hantaviral markers

Altogether 61 rodents were trapped: 7 *B. indica*, 27 *B. savilei*, 24 *Rattus exulans*, 1 *R. argentiventer*, 1 *R. rattus*, and 1 *R. norvegicus*. 53 lung tissue samples and 8 liver tissue samples have been collected and stored frozen until analysis. Screening by immunoblotting for the presence of hantaviral N-antigen using immunoblotting with anti-Dobrava virus antiserum revealed that 12 samples were considered positive or probably positive. A confirmatory immunoblotting was done with the anti-SEOV antiserum collected from *R. norvegicus *trapped in Indonesia [17]. Eight rodents were not confirmed as N-antigen-positive; these samples were subjected to the RT-PCR but none was found positive. Other four samples, all from *B. indica*, were confirmed positive for the hantaviral N-antigen. Two were trapped in Nakhon Pathom province, the other two in Nakhon Ratchasima province. The four N-antigen- positive rodents were analysed by RT-nested PCR and all were found positive for the hantaviral S- and M-segment nucleotide sequences.

Corresponding wild-type THAIV strains were designated as: THAIV/NakhonPathom/Bi0016/2004, THAIV/NakhonPathom/Bi0067/2004, THAIV/NakhonRatchasima/Bi0024/2004, and THAIV/NakhonRatchasima/Bi0017/2004. In the following: our wild-type strains refer to Thai0016, Thai0067, Thai0024, and Thai0017, respectively.

### Genetic analysis

Partial M segment sequences (nt 2021–2303) recovered from samples Thai0016 and Thai0067 were identical. Other three sequences differed at 3–7 positions, i.e. shown 1.1–2.4% diversity. Notably, all but one mutation were silent; strain Thai0067 had a homologous substitution of isoleucine to valine at pos 110 of the deduced sequence of the GnGc protein. This suggested a strong stabilising selection operating on the protein level. The M segment sequences of strains Thai0016 (Thai0067), Thai0024, and Thai0017 were most closely related to M-sequences of other hantaviruses carried by Murinae rodents. As expected, the highest level of identity was observed to the published M segment sequence of the THAIV isolate 749 originated from *B. indica *trapped in Thailand [8], 96–98%. The sequence identity to SEOV M-sequences was a bit lower, 73–78%, and the sequence identity to HTNV, DOBV and SAAV M-sequences was even lower, 68–74%. The M segment sequences of hantaviruses associated with Arvicolinae or Sigmodontinae rodents were most distant (identity of 59–68%).

Partial S segment sequences (nt 389–946) of four wild-type THAIV strains differed at 2–10 positions, i.e. showed 0.4–1.8% diversity. All nucleotide susbtitutions were silent suggesting, again, a strong stabilising selective pressure applied on the encoded part of the N protein (aa residues 110–300). The S-sequences of strains Thai0016 and Thai0067 differed at three positions thus confirming that the two strains are distinct. Four THAIV S-sequences showed high level of identity to SEOV, HTNV (also the HTNV-like DBSV and AMRV), DOBV, and SAAV S-sequences, 69%–75%. The S segment sequences recovered from *R. rattus*, which were trapped in Cambodia, showed the highest level of identity, 83–84%, with the newly recovered THAIV S-sequences.

From the rodent sample Thai0017 we were able to RT-amplify complete S segment sequence. It appeared to be 1882 nt in length (the first and the last 22 nucleotides from the complete S-amplicons originated from the PCR primer and therefore were not determined directly). The sequence consists of the 5'- (positive sense) non-coding region (NCR) of 46nt, the open reading frame of 1290 nt for the N protein (429 aa residues), and the 3'NCR of 546 nt. The deduced aa sequence of the THAIV N protein showed the highest identity (87%) to the N protein of SEOV. The N protein sequences of other Murinae-associated hantaviruses were less related: HTNV- 85%, DOBV – 83%, and SAAV – 82% while the N protein sequences of Arvicolinae- and Signodontinae- associated hantaviruses showed the lowest level of sequence identity: e.g., PUUV- 64% and SNV – 64%.

A comparison of our newly recovered wild-type THAIV S-sequence (Thai0017) and the sequence from the cell culture isolate 741 (Thai741) recently deposited to GenBank (Acc. number AB186420), showed that they are almost identical in length (1882 vs 1884 nt) and exhibit an overall diversity of 3.5%. The 5'-NCR of the Thai0017 strain is one nt longer while the 3'-NCR is 2 nt shorter than the corresponding regions of the Thai741 strain. The coding regions if the two strains show 3.2% diversity and the NCRs show 3.8% diversity. Deduced N protein sequences are 98.8% identical and all five substitutions, L39F, R41K, R73K, M226V, and I322V are homologous. This once again stresses the point that the N protein sequence is highly conserved within a given hantavirus type due to functional constrains (see, e.g., [22,23]).

### Phylogenetic analysis

**Figure 1:**
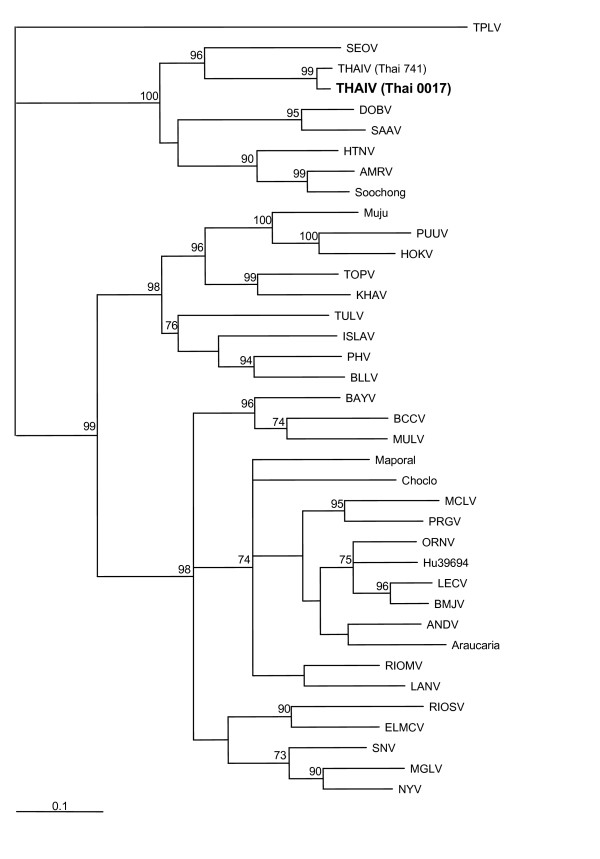
Phylogenetic tree (ML-TreePuzzle) of hantaviruses based on the complete coding region of the S segment. Only bootstrap support values greater than 70% are shown. *Complete S-segment sequences:*Thottapalayam virus (TPLV) (GeneBank accession no. AY526097); Seoul virus (SEOV), strain SR11 (M34881); Thailand virus (THAIV), strain 741 (AB186420); Dobrava virus (DOBV), strain Dobrava (L41916); Saaremaa virus (SAAV), strain Saaremaa/160v (AJ009773); Hantaan virus (HTNV), strain 76–118 (M14626); Amur virus (AMRV), strain Solovey/AP63/1999 (AB071184); Soochong virus, strain SC-1 (AY675349); Muju virus, strain Muju99-28 (DQ138142); Puumala virus (PUUV), strain Sotkamo (X61035); Hokkaido virus (HOKV), strain Kamiiso-8-Cr-95 (AB010730); Topografov virus (TOPV), strain Ls136V (AJ011646); Khabarovsk virus (KHAV), strain MF-43 (U35255); Tula virus (TULV), strain Moravia/02v (Z69991); Isla Vista virus (ISLAV), strain MC-SB-47 (U19302); Prospect Hill virus (PHV), strain PH-1 (Z49098); Bloodland lake virus (BLLV), strain MO46 (U19303); Bayou virus (BAYV), strain Louisiana (L36929); Black Creek Canal (BCCV) (L39949); Muleshoe virus (MULV), strain SH-Tx-339 (U54575); Maporal virus, strain HV-97021050 (AY267347); Choclo virus (DQ285046); Maciel virus (MCLV), strain 13796 (AF482716); Pergamino virus (PRGV), strain 14403 (AF482717); Oran virus (ORNV), strain 22996 (AF482715); Hu39694 virus (AF482711); Lechiguanas virus (LECV), strain 22819 (AF482714); Bermejo virus (BMJV), strain Oc22531 (AF482713); Andes virus (ANDV), strain AH-1 (AF324902); Araucaria virus, strain HPR/02-72 (AY740625); Rio Mamore virus (RIOMV), strain Om-556 (U52136); Laguna Negra virus (LANV), strain 510B (AF005727); Rio Segundo virus (RIOSV), strain RMx-Costa-1 (U18100); El Moro Canyon (ELMCV), strain RM-97 (U11427); Sin Nombre virus (SNV), strain NM H10 (L25784); Monongahela virus (MGLV), strain Monongahela-1 (U32591); and New York virus (NYV), strain RI-1 (U09488).

**Figure 2:**
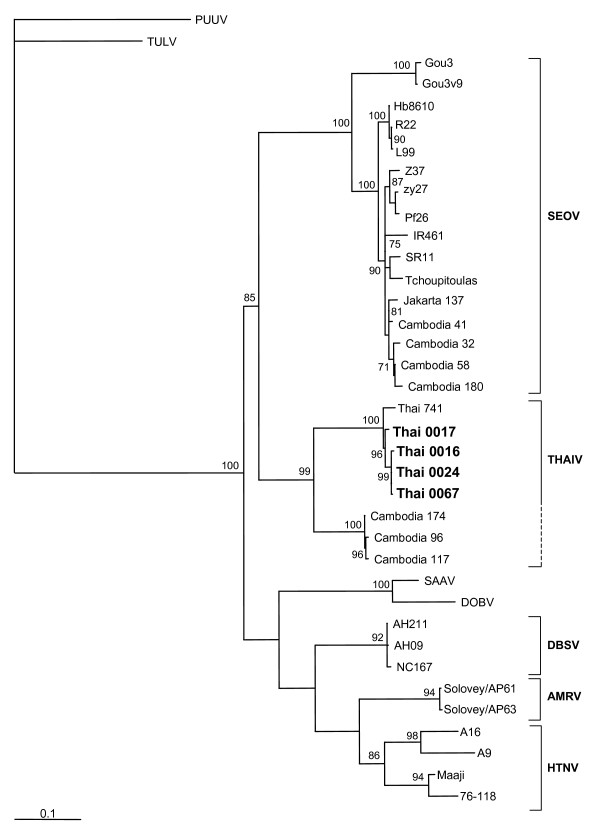
Phylogenetic tree (ML-TreePuzzle) of hantaviruses based on partial sequence (nt 389–946) of the S segment. Only bootstrap support values greater than 70% are shown. *Partial S-segment sequences:*PUUV, strain Sotkamo (X61035); TULV, strain Moravia/02v (Z69991); SEOV, strains Gou3 (AF184988), Gou3v9 (AB027522), Hb8610 (AF288643), R22 (AF288295), L99 (AF288299), Z37 (AF187082), zy27 (AF406965), Pf26 (AY006465), IR461 (AF329388), SR11 (M34881), Tchoupitoulas (AF329389), Jakarta137 (AJ620583), Cambodia (Camb)41 (AJ427501), Camb32 (AJ427508), Camb58 (AJ427510), Camb180 (AJ427506), Camb174 (AJ427513), Camb96 (AJ427512), and Camb117 (AJ427511); THAIV virus, strain 741 (AB186420); SAAV, strain Saaremaa/160v (AJ009773); DOBV, strain Dobrava (L41916); Da Bie Shan virus (DBSV), strains NC167 (AB027523), AH211 (AF288647), and AH09 (AF285264); Amur virus (AMRV), strains Solovey/AP63/1999 (AB071184), and Solovey/AP61/1999 (AB071183); and HTNV, strains A16 (AB027099), A9 (AF329390), Maaji (AF321095), and 76–118 (M14626).

**Figure 3:**
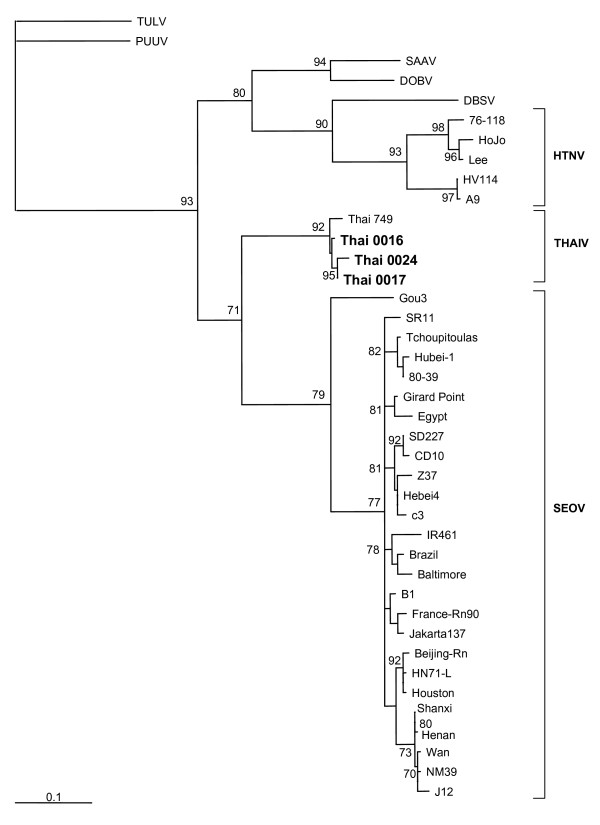
Phylogenetic tree (Fitch-Margoiliash) of hantaviruses based on partial sequence (nt 2021–2303) of the M segment. Only bootstrap support values greater than 70% are shown. *Partial M-segment sequences:*PUUV, strain Sotkamo (X61034); TULV, strain Moravia/02v (Z69993); DOBV, strain Dobrava (L33685); SAAV, strain Saaremaa/160v (AJ009774); DBSV, strain NC167 (AB027115); HTNV, strains 76–118 (M14627), HoJo (D00376), Lee (D00377), HV114 (L08753), and A9 (AF035831); THAIV, strain 749 (L08756); and SEOV, strains Gou3 (AB027521), SR11 (M34882), Tchoupitoulas (U00473), Hubei-1 (S72343), 80–39 (S47716), Girard Point (U00464), Egypt (U00463), SD227 (AB027091), CD10 (AB027092), Z37 (AF187081), Hebei4 (AB027089), c3 (AB027088), IR461 (AF458104), Brazil (U00460), Baltimore (U00151), B1 (X53861), France-Rn90 (AJ878418), Jakarta137 (AJ620583), Beijing-Rn (AB027087), HN71-L (AB027085), Houston (U00465), Shanxi (AB027084), Henan (AB027083), Wan (AB027081), NM39 (AB027080), and J12 (AB027082).

On the phylogenetic trees constructed for complete and partial S segment sequences and also for partial M-segment sequences THAIV strains clustered together and formed a well supported group. Same branching pattern was seen on the trees calculated using different algorithms; the ML-Puzzle-trees are shown on Figures 1 to 3. Not surprisingly, THAIV sequences were placed within the group of Murinae-associated hantaviruses and shared a recent common ancestor with SEOV reflecting a close relationships between *Bandicota *and *Rattus *genera. These two hantavirus species formed a sister taxa to another group that included hantaviruses associated with *Apodemus *mice: DOBV, SAAV, HTNV and also HTNV-like viruses Da Bie Sha, and Amur/Soochong. Within the group of THAIV strains, some signs of geographical clustering were seen. On the partial M-segment tree, the sequences of wt-strains from Nakhon Ratchasima province (Thai0024 and Thai0017) were separated from the sequence of Thai0016 and Thai0067 strains (Nakhon Pathom province). On both partial S- and partial M- segment trees the wt-strains from Nakhon Pathom and Nakhon Ratchasima were separated from the isolates Thai741 and Thai749.

Most notably, the phylogenies inferred for the partial S segment sequences revealed a well-supported monophily of THAIV strains and wt-strains associated with *R. rattus *in Cambodia [described by Reynes et al., 2003 [9]]. These two clusters of strains were clearly separated from the major cluster of SEOV strains including *R. rattus*-associated strain Gou originated from Zhejiang (China) [24]. This result suggested that there are two distinct hantaviral types found in *R. rattus*: "Cambodia-like" (a close relative of THAIV) and "China-like" (Gou, a close relative of *bona fide *SEOV).

## Discussion

### Rodent hosts for hantaviruses in Thailand

Our data confirmed hantavirus circulation in at least two provinces of Thailand: Nakhon Pathom and Nakhon Ratchasima. Notably, four *B. indica *rodents were found hantavirus-positive but none of *B. salivei *suggesting *B. indica *as a primary host for THAIV. *Rattus *species were all found hantavirus-negative during this study. However previous serological investigations of hantaviruses in Thailand have shown other rodents as possible vectors: *Rattus rattus *[12,25,26], *R. exulans *[11,26,27]; *R. norvegicus *[11,12,27] and *R. losea *[26]. A more intensive study is needed to clarify this issue.

### Results of (phylo)genetic analyses of THAIV and related viruses

In this paper, for the first time, the complete S segment sequence of THAI virus is described. The new genetic information is in line with our previous knowledge based on the complete M segment sequence: THAIV is a distinct hantavirus species that shows a substantial genetic diversity from other members of the *Hantavirus *genus and shares the most recent common ancestor with SEOV and the more ancient common ancestor with other Murinae-associated viruses. Four newly described wt- strains of THAIV showed decent genetic diversity between themselves, 0.4–2.4%, and also to the previously described THAIV isolate (2–4%, in the partial M.-segment sequence). Interestingly, these wt strains, which originated from two trapping areas 250 km apart, showed some signs of geographical clustering, the feature shared by all known hantaviruses except the "cosmopolitan" SEOV associated with *R. norvegicus *[4,5].

When analysing the partial S segment sequences we observed that the newly described THAIV strains are monophyletic with the wt hantavirus strains associated with *R. rattus *in Cambodia. These two sister taxa are separated from SEOV strains associated with *R. norvegicus *worldwide but also from the *R. rattus*-associated strain Gou originated from China. This phylogeny is different from the phylogeny inferred by Reynes et al [9] for partial S segment sequence (nt 370–970): in the later, the THAIV sequence (Thai749) is not monophyletic with any *Rattus- *associated virus but instead occupies the most ancestral node in the THAIV-HTNV-DOBV-SAAV-SEOV clade.

Reynes and co-authors [9] suggested that at least two subtypes of SEOV carried by *R. rattus *circulate in Asia. Phylogeny presented in this paper (Fig. 2) suggests that there might be two distinct hantaviruses associated with *R. rattus*. The first of them, Gou virus, is either a subtype of SEOV or a closely related to SEOV but distinct hantavirus. The second hantavirus, which was found in Cambodia, is a relative of THAIV but a distinct entity as well. Further investigation is needed to unwrap this intriguing story. For instance, it might be worth studying whether the "Cambodia virus" is a product of a host-switch of pre-THAI from *Bandicota *to *Rattus*.

The results of previous studies suggested that new viruses, different hosts and different human syndromes may be expected to be discovered in the future in Southeastern Asia where Muridae rodents are endemic and highly diversified and where the human population is regularly exposed to them. The recent discovery of a new hantavirus in Guinea [28] demonstrate that hantaviruses have to be tracked wherever Muridae rodents are living. Further studies are needed to assess the reality of an endemic Southeast Asian group of hantaviruses and to understand their particularities, their current distribution among rodents in different areas and in different landscapes and finally their potential dangerousness for humans. This also supposes the improvement of our knowledge of the ecology and biogeography of the hantavirus natural reservoirs in Southeast Asia. Thailand, which health system is strongly organized and possesses important and detailed archives has all the necessary resources to organize such a program. The results may be of interest for all the surrounding countries and give rise to a regional cooperation in this field of study.

Most recently we became aware of the manuscript of S. Pattamadilok and co-authors [29] in which they characterized the S segment sequence recovered from the THAIV isolate and also performed antigenic cross-reactivity studies of rodent and human sera collected in Thailand. Their observations on THAIV-positive bandicoot rats as well as results of the phylogenetic analyses are nicely in line with our data reported here. Most interestingly, the serum of one patient with the HFRS symptoms showed high titers of THAIV-neutralisiung antibodies suggesting that this hantavirus is a human pathogen.

## Authors' contributions

**JPH **participated in the study design and coordination, trapping and screening of rodents, and drafting the manuscript. **AngP **participated in the screening of the rodent samples, performed RNA isolation, RT-PCR and sequencing, participated also in the genetic analysis and drafting the manuscript. **VH **participated in the study design, trapping and screening of rodents, and drafting the manuscript. **KN **participated in (phylo)genetis analyses and drafting the manuscript. **JL **participated in the study coordination and screening of rodents. **ÅL **participated in the study coordination and drafting the manuscript. **YS **participated in the study coordination and trapping and screening of rodents. **HH **participated in the study design and coordination and drafting the manuscript. **AP **participated in the study design and coordination, (phylo)genetic analyses and drafted the manuscript. All authors read and approved the final manuscript.
